# Coexisting Sarcoid Reaction and Sarcoidosis Requiring Differentiation from Pancreatic Adenocarcinoma Recurrence: A Case Report

**DOI:** 10.70352/scrj.cr.25-0670

**Published:** 2026-04-10

**Authors:** Ryo Horie, Makoto Takahashi, Tatsuya Hayashi, Ryuji Takada, Taiki Tsuji, Taku Higashihara, Hideyuki Horike, Wataru Yamagata, Akihiko Wada, Naoto Yokogawa, Sachiko Izumi, Junko Araki, Haruka Okada, Iichiroh Onishi, Yasuhiro Morita

**Affiliations:** 1 Department of General Surgery, Tokyo Metropolitan Tama Medical Center, Tokyo, Japan; 2 Department of Gastroenterology, Tokyo Metropolitan Tama Medical Center, Tokyo, Japan; 3 Department of Respiratory Medicine, Tokyo Metropolitan Tama Medical Center, Tokyo, Japan; 4 Department of Rheumatology, Tokyo Metropolitan Tama Medical Center, Tokyo, Japan; 5 Department of Radiology, Tokyo Metropolitan Tama Medical Center, Tokyo, Japan; 6 Department of Pathology, Tokyo Metropolitan Tama Medical Center, Tokyo, Japan; 7 Department of Pathology, Institute of Science Tokyo Hospital, Tokyo, Japan

**Keywords:** pancreatic ductal adenocarcinoma, postoperative recurrence, sarcoid reaction, sarcoidosis, *Propionibacterium acnes*-specific antibody staining

## Abstract

**INTRODUCTION:**

Pancreatic ductal adenocarcinoma (PDAC) is one of the most severe malignant tumors. The recurrence rate is high, even after radical surgery. A sarcoid reaction is a histological finding that occurs in the lymph nodes in response to malignant tumors, infections, or foreign substances. Its histopathology is similar to that of sarcoidosis. Here, we report a case of coexisting sarcoid reaction and sarcoidosis that required differentiation from PDAC recurrence.

**CASE PRESENTATION:**

A 65-year-old woman was diagnosed with locally advanced PDAC. She underwent modified FOLFIRINOX (folinic acid, fluorouracil, irinotecan, and oxaliplatin) therapy for 6 months, after which the tumor shrunk, and there were no new metastatic lesions. Additionally, she underwent chemoradiation therapy (50 G/25 Fr and S-1), and a distal pancreatectomy with en bloc celiac axis resection and splenectomy were performed. Pathological diagnosis was PDAC, T2N0M0. Pathological findings of the regional lymph nodes showed no malignancy and noncaseating epithelioid cell granulomas, which were thought to be a sarcoid reaction. She received S-1 therapy for 6 months as adjuvant chemotherapy. On contrast-enhanced CT 1 year after the operation, enlarged para-aortic lymph nodes and multiple grainy pulmonary shadows were detected. Although recurrence was suspected, tumor marker levels did not increase. PET-CT revealed fluorodeoxyglucose accumulation in several lymph nodes, such as the para-aortic or neck, as well as in the bilateral femoral muscles. No accumulation was observed in the lung parenchyma. These findings were atypical of postoperative recurrence. Endoscopic ultrasonography-guided lymph nodes and body-surface ultrasonography-guided femoral muscle biopsies were performed. No malignancy was found in all the specimens, and noncaseating epithelioid cell granulomas were found. *Propionibacterium acnes*-specific antibody staining showed that the regional lymph nodes removed during surgery were negative, and the para-aortic lymph nodes and femoral muscles were positive. The patient was diagnosed with a sarcoid reaction to PDAC and muscular sarcoidosis. The patient remained recurrence-free for 3 years and 2 months postoperatively.

**CONCLUSIONS:**

When enlarged lymph nodes are detected after surgery for a malignant tumor, we have to consider recurrence at first; however, it is important to make careful assessments because other factors are occasionally present.

## Abbreviations

ACE
: angiotensin-converting enzyme
CA19-9
: carbohydrate antigen 19-9
DP-CAR
: distal pancreatectomy with en bloc celiac axis resection and splenectomy
DUPAN-2
: duodenal pancreatic cancer antigen-2
EUS-TA
: endoscopic US-guided tissue acquisition
FDG
: fluorodeoxyglucose
FOLFIRINOX
: folinic acid, fluorouracil, irinotecan, and oxaliplatin
PAB
: *Propionibacterium acnes*-specific antibody
PDAC
: pancreatic ductal adenocarcinoma
S-1
: tegafur/gimeracil/oteracil
sIL-2R
: soluble interleukin-2 receptor

## INTRODUCTION

Owing to advances in preoperative therapy,^1)^ surgery,^2)^ and adjuvant therapy,^3)^ the prognosis of PDAC is improving^4)^; however, it remains one of the most severe prognostic malignant tumors. Surgical resection is the only available curative therapy for PDAC. The recurrence rate remains high, and the prognosis is poor.^5,6)^

A sarcoid reaction is a histological finding that occurs in the lymph nodes in response to malignant tumors, infections, or foreign substances. The pathological finding is a noncaseating epithelioid cell granuloma similar to that of sarcoidosis. Sarcoid reactions occur in patients with malignant tumors.^7)^ Although it is difficult to differentiate sarcoid reaction from sarcoidosis pathologically, PAB staining has been reported to be useful.^8)^ We encountered a case of coexisting sarcoid reaction and sarcoidosis, which was detected as enlarged regional lymph nodes and multiple pulmonary grainy shadows 1 year after a radical procedure for PDAC, requiring differentiation from recurrence.

## CASE PRESENTATION

**Fig. 1:**
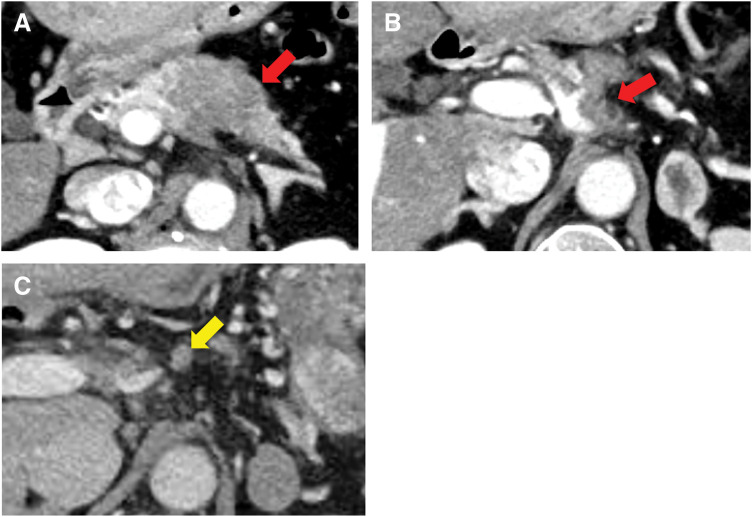
(**A**) Contrast-enhanced CT showing a 38-mm hypovascular tumor (red arrow) in the body of the pancreas. (**B**) The tumor had invaded the celiac (red arrow), splenic, and common hepatic arteries. (**C**) Before treatment, the regional lymph nodes appeared slightly prominent (yellow arrow).

In November 2021, a 65-year-old woman with a medical history of hypertension had an abnormal blood glucose level and a 40-mm mass in the corpus of the pancreas on abdominal ultrasonography performed by her primary care physician. Contrast-enhanced CT revealed pancreatic body cancer with invasion of the celiac, common hepatic, and splenic arteries and backward organization, and slightly prominent regional lymph nodes (**Fig. 1A**–**1C**).

**Fig. 2:**
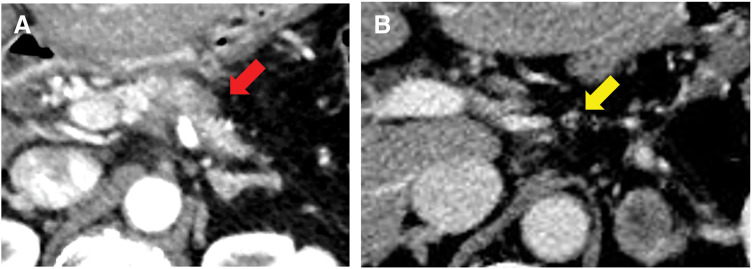
(**A**) After chemotherapy, the tumor shrank to a size of 20 mm (red arrow). (**B**) After chemotherapy, the regional lymph nodes showed slight shrinkage (yellow arrow).

**Fig. 3:**
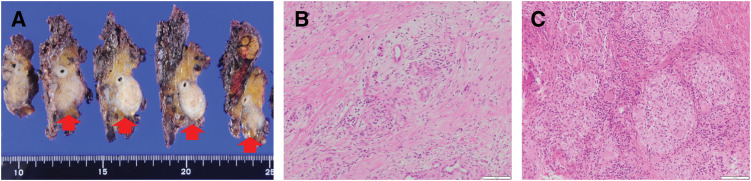
(**A**) A tumor measuring 37 × 37 × 20 mm was identified in the body of the pancreas (red arrows). (**B**) Microscopically, atypical cells with prominent nucleoli and a round to oval shape forming irregular gland-like structures, proliferating against a background of fibrosis. Scale bar: 100 µm. (**C**) No malignant findings were observed in the resected lymph nodes. Noncaseating epithelioid cell granulomas with multinucleated giant cells were identified, suggesting a sarcoid reaction. Scale bar: 100 µm.

She was referred to a gastroenterologist at our hospital, and her blood test results were as follows: CA19-9 was 92.5 U/mL (normal, <37.0 U/mL) and DUPAN-2 was 1200 U/mL (normal, <150 U/mL). She underwent EUS-TA and was diagnosed with an unresectable locally advanced pancreatic body adenocarcinoma without metastasis. She started chemotherapy with modified FOLFIRINOX for 6 months, which resulted in tumor size reduction to 20 mm without metastasis (**Fig. 2A**) and slight shrinkage of the regional lymph nodes (**Fig. 2B**), and a decrease in the tumor markers (CA19-9, 22.6 U/mL; DUPAN-2, 210 U/mL). The tumor was assessed to be resectable, and she was referred to the Department of General Surgery. She also underwent radiation therapy (50 G/25 Fr) with S-1 for 1 month. A radical operation (DP-CAR) was performed in August 2022. Resection margins were negative, and regional lymph nodes were resected (**Fig. 3A** and **3B**). Pathological findings of the lymph nodes showed no malignancy and noncaseating epithelioid cell granulomas, which were thought to be a sarcoid reaction (**Fig. 3C**). According to the 8th edition of the Union for International Cancer Control, the pathological diagnosis was PDAC, T2N0M0, stage IB. Most pancreatic cancer cells underwent degeneration; however, a few viable tumor cells remained. Thus, the therapeutic effect was classified as grade 3 according to the Evans grading system.^9)^ Postoperatively, the patient underwent adjuvant chemotherapy with S-1 for 6 months.

**Fig. 4:**
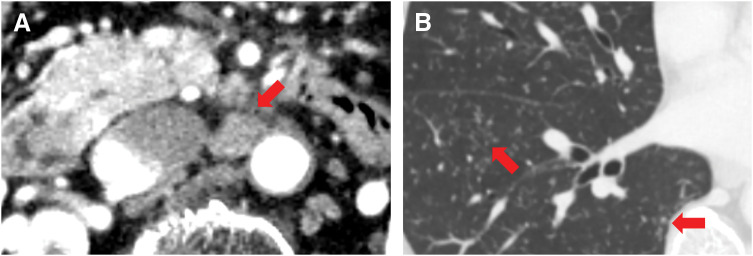
(**A**) CT image taken 1 year postoperatively showing enlargement of the para-aortic lymph nodes (red arrow). (**B**) CT taken 1 year postoperatively showing multiple grainy shadows in the lungs (red arrows).

**Fig. 5:**
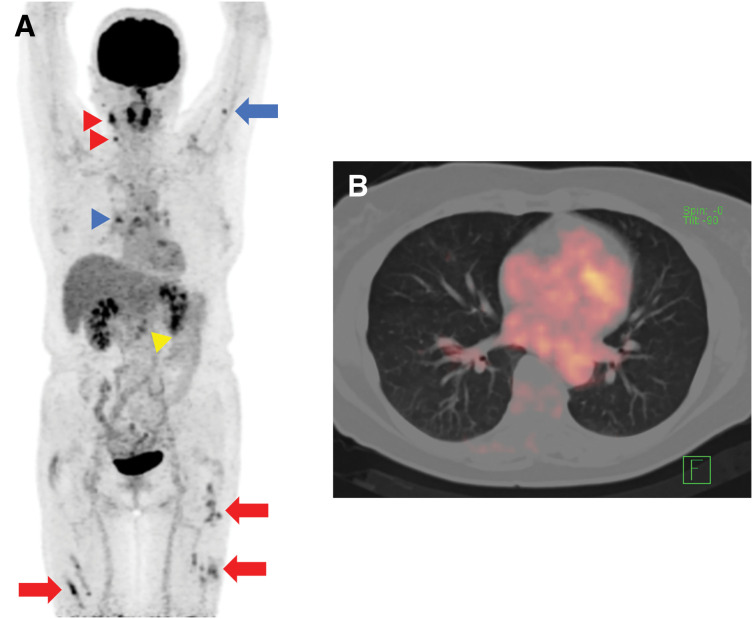
(**A**) On PET-CT, there were FDG accumulations at the cervical lymph nodes (red arrowheads), left upper arm (blue arrow), mediastinum and hilus (blue arrowhead), para-aortic lymph nodes (yellow arrowhead), and bilateral femoral muscles (red arrows). (**B**) There was no FDG uptake in the lung parenchyma. FDG, fluorodeoxyglucose

**Fig. 6:**
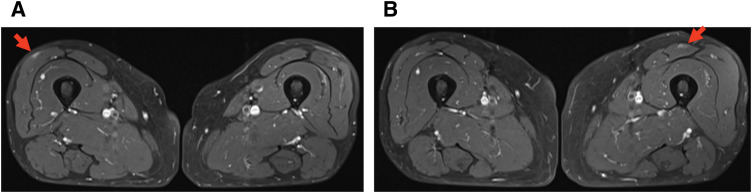
(**A**) Contrast-enhanced MRI showing a nodule with uniform enhancement in the right vastus lateralis muscle (red arrow). (**B**) Contrast-enhanced MRI showing a nodule with uniform enhancement in the left rectus femoris muscle (red arrow).

**Fig. 7:**
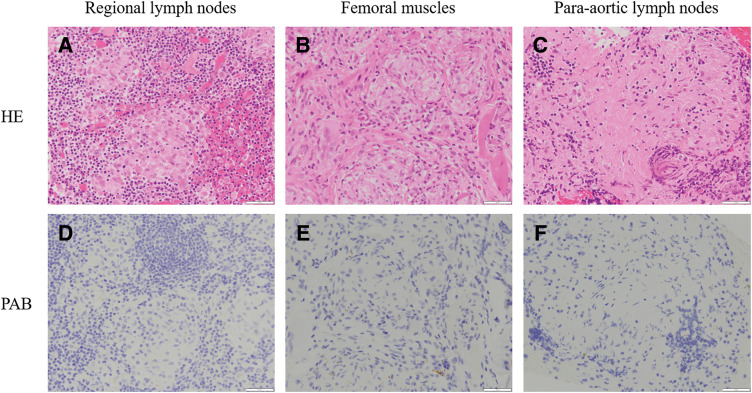
(**A**) HE staining of the regional lymph nodes collected during surgery showing smaller granulomas with few multinucleated giant cells. Scale bar: 50 μm. (**B**) HE staining of the femoral muscles showing larger granulomas with prominent multinucleated giant cells. Scale bar: 50 μm. (**C**) HE staining of the para-aortic lymph nodes showing larger granulomas with prominent multinucleated giant cells and asteroid bodies. Scale bar: 50 μm. (**D**) PAB staining, specific for *P. acnes*, showing negative results in the regional lymph nodes collected during surgery. Scale bar: 50 μm. (**E**) PAB staining showing positive results in the femoral muscles. Scale bar: 50 μm. (**F**) PAB staining showing positive results in the para-aortic lymph nodes. Scale bar: 50 μm. HE, Hematoxylin and eosin; PAB, *Propionibacterium acnes*-specific antibody

In August 2023, 1 year after surgery, enlarged para-aortic lymph nodes and multiple pulmonary grainy shadows were found on follow-up contrast-enhanced CT (**Fig. 4A** and **4B**). Although recurrence and PDAC metastasis were considered, tumor marker levels remained low (CA19-9, 12.1 U/mL; DUPAN-2, 66 U/mL). On PET-CT, there were FDG accumulations at neck lymph nodes, left upper arm, mediastinum and hilus, para-aortic lymph nodes, and bilateral femoral muscles (**Fig. 5A**); however, there were no lung parenchymal accumulations (**Fig. 5B**). Nonneoplastic accumulations, such as chronic granulomatous, reactive, or inflammatory, were considered rather than recurrences. We concluded that these results were not typical of the postoperative recurrence of PDAC. First, we tried a biopsy of the neck lymph nodes; however, the vessels were so close that the biopsy was abandoned. Contrast-enhanced MRI revealed nodules with uniform enhancement in the right vastus lateralis and left rectus femoris muscles, suggesting the possibility of an inflammatory disease, such as sarcoidosis (**Fig. 6A** and **6B**). EUS-TA of the para-aortic lymph nodes and body surface US biopsy of the femoral muscles were performed. None of the specimens showed features of malignancy, but showed noncaseating epithelioid cell granulomas, as seen in the regional lymph nodes resected during the surgery. However, the regional lymph nodes resected during surgery exhibited smaller granulomas and a few multinucleated giant cells (**Fig. 7A**), whereas the specimens from the quadriceps femoris (**Fig. 7B**) and para-aortic lymph nodes (**Fig. 7C**) displayed larger granulomas with prominent multinucleated giant cells. Additionally, asteroid bodies were observed in the para-aortic lymph nodes (**Fig. 7C**).

Sarcoidosis markers were not elevated (ACE, 18.9 U/L; sIL-2R, 478 U/mL). The patient did not exhibit any heart or eye symptoms. PAB staining of the regional lymph nodes resected during surgery showed negative results (**Fig. 7D**), whereas staining of the femoral muscle and para-aortic lymph node specimens showed positive results (**Fig. 7E** and **7F**). These results led us to a diagnosis of sarcoid reaction preoperatively and postoperative muscular sarcoidosis. However, it was determined that the condition was not a recurrence of PDAC.

**Fig. 8:**
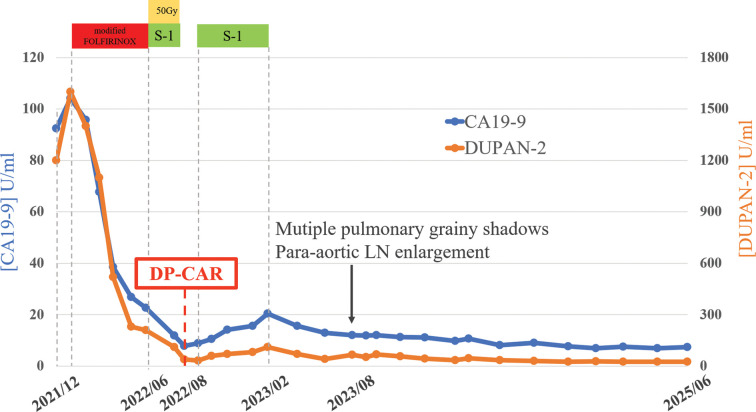
Clinical course of this case. Three years have passed since the radical surgery, and the tumor markers have remained at a low value. CA19-9, carbohydrate antigen 19-9; DP-CAR, distal pancreatectomy with en bloc celiac axis resection and splenectomy; DUPAN-2, duodenal pancreatic cancer antigen-2; FOLFIRINOX, folinic acid, fluorouracil, irinotecan, and oxaliplatin; LN, lymph node; S-1, tegafur/gimeracil/oteracil

The patient has been asymptomatic and recurrence-free for 3 years and 2 months postoperatively. The patient’s clinical course is shown in **Fig. 8**.

## DISCUSSION

PDAC is a highly aggressive malignancy with a high recurrence rate even after curative resection.^5,6)^ Although surgical resection remains the only potentially curative treatment, postoperative surveillance is crucial because of frequent recurrences. In this context, distinguishing true recurrence from benign conditions such as sarcoid reactions or sarcoidosis is essential to avoid misdiagnosis and unnecessary treatment.

Differentiating it from systemic disease of sarcoidosis, noncaseating epithelioid cell granulomas reacting to malignant tumors or foreign substances are called sarcoid reaction or sarcoid-like reaction.^10)^ Sarcoid reaction is a biological defense mechanism observed in regional lymph nodes.^11)^ The relationship between malignant tumors and sarcoid reaction was described in the 1910s. Malignant tumors that produce sarcoid reaction have been reported in various cancers.^12–14)^ However, there are few reports of sarcoid reactions complicating PDAC. Marianna and co-workers reported a case of PDAC with sarcoidosis/sarcoid reaction.^15)^

In addition to malignant tumors, infections or antitumor drugs are thought to be the causes of sarcoid reaction. Although numerous cases of sarcoid reactions induced by immune checkpoint inhibitors have been described,^16)^ such reactions associated with cytotoxic agents like FOLFIRINOX or S-1 have not been documented.

There are several theories regarding the causes of sarcoid reactions associated with cancer. Brincker proposed that certain factors that leak from cancer cells reach the lymph nodes during lymphatic flow and form granulomas.^17)^ Gorton and Linell reported the reaction of lymph nodes to metabolites or decaying substances in cancer.^10)^ There are also theories that suggest sarcoid reactions are associated with tumor immunity and good prognosis.^18)^ In contrast, Huh et al. reported no such relationship.^7)^ In this case, the patient has been recurrence-free for 3 years and 2 months after the radical surgery.

One year postoperatively, CT revealed enlarged para-aortic lymph nodes and grainy pulmonary shadows, raising a suspicion of PDAC recurrence. Owing to the absence of elevated tumor markers and atypical FDG uptake sites on PET-CT (e.g., bilateral femoral muscles) for PDAC recurrence, EUS-TA of the para-aortic lymph nodes and biopsy of the femoral muscles were performed to obtain a definitive histological diagnosis. No findings suggestive of PDAC recurrence were observed in the para-aortic lymph nodes or femoral muscles, and noncaseating epithelioid cell granulomas were identified.

First, the noncaseating epithelioid cell granulomas observed in the para-aortic lymph nodes and bilateral femoral muscles were considered to be caused by sarcoid reactions, similar to those observed in the lymph nodes collected during the surgery. However, there were some differences in the histopathological findings. The regional lymph nodes sampled during the surgery exhibited smaller granulomas and a few multinucleated giant cells, whereas specimens from the quadriceps femoris and para-aortic lymph nodes displayed larger granulomas with prominent multinucleated giant cells. The latter is not a pathological characteristic of sarcoid reaction, but rather of sarcoidosis.

Kinoshita et al. reported differences in granuloma size and the number of multinucleated cells between sarcoid reaction and sarcoidosis.^8)^ Sarcoidosis had larger granulomas (0.05 ± 0.04 vs. 0.02 ± 0.01 mm^2^, p <0.01) and a larger number of multinucleated cells (0.58 ± 0.75 vs. 0.02 ± 0.04/mm^2^, p <0.01) than sarcoid reaction. In this case, the size of the granulomas and number of multinucleated cells in the quadriceps sample were larger than those in the surgical regional lymph nodes.

To further investigate these differences, we performed PAB staining specific for *P. acnes* on each specimen. A relationship between sarcoidosis and *P. acnes* has been indicated, which sometimes helps in differentiation. Kinoshita et al. showed that *P. acnes*-specific monoclonal antibody reactivity is more frequent in a sarcoidosis group than in a sarcoid reaction group (82.4% vs. 12.5%, p <0.01).^8)^ In this case, the results showed that the regional lymph nodes resected during the surgery were negative (**Fig. 7D**). In contrast, the quadriceps and para-aortic lymph nodes were positive (**Fig. 7E** and **7F**).

Because sarcoid reaction and sarcoidosis are also hypermetabolic, PET-CT may show abnormal FDG accumulation,^19,20)^ which may result in false-positive results for the evaluation of postoperative recurrence. There is also a case report of lung cancer in which FDG accumulation in bilateral hilar and mediastinal lymph nodes was detected after induction of chemotherapy, which revealed noncaseating epithelioid cell granulomas on biopsy, resulting in a diagnosis of sarcoidosis.^21)^ Another case of lung cancer showed pathological N2 metastases on preoperative biopsy, and the tumor shrank with chemotherapy; however, PET-CT showed abnormal accumulation at the hilar lymph nodes, resulting in a sarcoid reaction postoperatively.^22)^ Kang et al. argued that PET-CT findings of new hypermetabolic bilateral hilar and mediastinal lymph nodes in the absence of uptake at primary tumor sites and extrathoracic areas may be highly suggestive of sarcoid reaction and that biopsy is essential to differentiate between sarcoid reaction and disease progression of malignancy.^23)^ In this case, PET-CT showed abnormal FDG accumulation at the para-aortic lymph nodes, and recurrence was suspected; however, abnormal accumulation in the bilateral femoral muscles led us to consider the possibility of a false-positive result. We assessed that the recurrence was negative because biopsies of the para-aortic lymph nodes and femoral muscles did not show evidence of malignancy.

Although sarcoid reaction generally forms noncaseating epithelioid cell granulomas in the vicinity of malignant tumors or in the regional lymph nodes, there have been no reported cases of similar pathology in the bilateral femoral muscles and enlarged cervical lymph nodes, with abnormal FDG accumulation, as in this case.

These results suggest that the patient developed a sarcoid reaction preoperatively and that she developed muscular sarcoidosis postoperatively.

Regarding the sarcoid reaction, it is natural to consider the origin to be PDAC. The time of appearance of muscular sarcoidosis was unknown because PET-CT was not performed preoperatively, and the patient was asymptomatic.

A retrospective review revealed that small pulmonary lesions were present on preoperative CT scans, suggesting that sarcoidosis may have existed before surgery. However, enlarged para-aortic and cervical lymph nodes were not observed preoperatively, indicating that sarcoidosis became clinically apparent postoperatively. The lesions in the para-aortic lymph nodes and femoral muscles were anatomically distant from the pancreas, and the morphology of the granulomas was markedly different from that of the regional lymph nodes resected during surgery. Therefore, these findings were considered indicative of sarcoidosis rather than a sarcoid reaction.

**Table 1** summarizes the key differences between sarcoid reactions and sarcoidosis and highlights their clinical, radiological, and pathological features.^7,8,17,19,20)^

**Table 1:** Comparison between sarcoid reaction and sarcoidosis

Feature	Sarcoid reaction	Sarcoidosis
Definition	Localized noncaseating granulomatous reaction, often adjacent to malignancy	Systemic granulomatous disease of unknown etiology involving multiple organs
Etiology	Immune-mediated reaction triggered by tumor antigens or therapy	Idiopathic; dysregulated immune response to unidentified antigen(s)
Organ involvement	Localized, typically regional lymph nodes draining a tumor	Multisystem involvement (lungs, lymph nodes, skin, eyes, etc.)
Clinical symptoms	Usually asymptomatic; incidental finding during cancer staging	Variable; respiratory, ocular, dermatologic, or systemic symptoms
Radiologic findings	Localized lymphadenopathy mimicking metastasis	Bilateral hilar lymphadenopathy, pulmonary infiltrates, extrapulmonary lesions
PET-CT findings	FDG uptake may mimic metastatic disease	FDG uptake is frequently observed in active granulomatous lesions
Histopathology	Noncaseating epithelioid granulomas located near tumor tissue; typically small, sparse, and with few multinucleated giant cells	Well-formed noncaseating granulomas with prominent multinucleated giant cells and occasional asteroid/Schaumann bodies; systemic distribution
PAB staining	Typically negative; *P. acnes* rarely detected	Often positive for *P. acnes* within granulomas
Laboratory findings	Usually normal; no systemic inflammatory markers	May show elevated ACE, sIL-2R, hypercalcemia, lymphopenia
Treatment	No specific treatment; resolves with treatment of the underlying malignancy	Corticosteroids or immunosuppressants when symptomatic or organ-threatening
Prognosis	Benign; no systemic progression	Variable; may be self-limiting, chronic, or progressive

In the present case, the regional lymph nodes resected during surgery were consistent with sarcoid reaction, whereas the postoperative lesions involving the para-aortic lymph nodes and bilateral thigh muscles were more compatible with systemic sarcoidosis.

ACE, angiotensin-converting enzyme; FDG fluorodeoxyglucose; PAB, *Propionibacterium acnes*-specific antibody; sIL-2R, soluble interleukin-2 receptor

In this case, the regional lymph nodes resected during surgery were consistent with a sarcoid reaction, whereas the postoperative lesions involving the para-aortic lymph nodes and bilateral femoral muscles were more compatible with systemic sarcoidosis.

This case is noteworthy for several reasons. First, it demonstrates a rare chronological and anatomical distinction between sarcoid reaction and systemic sarcoidosis, with the former identified intraoperatively and the latter emerging postoperatively. Second, it highlights a significant diagnostic challenge in the context of PDAC surveillance in which FDG-avid lesions in atypical locations can mimic recurrence. Third, PAB staining provided valuable histopathological insights supporting the diagnosis of sarcoidosis. These features underscore the importance of this case in illustrating the need for careful differential diagnosis to avoid misinterpretation of postoperative imaging findings.

## CONCLUSIONS

The present case involved coexisting sarcoid reaction and muscular sarcoidosis that required differentiation from PDAC recurrence. To the best of our knowledge, no cases of simultaneous sarcoidosis and sarcoid reactions arising during cancer treatment have been reported, as was observed in this case. Although enlargement of lymph nodes after surgery for a malignant tumor makes us consider recurrence at first, other findings must be evaluated carefully to determine whether recurrence has occurred. Careful differential diagnosis is essential because misdiagnosis may lead to unnecessary treatment.

## Declarations

### Availability of data and materials

Data sharing is not applicable to this article.
